# Considerations for and Guidance to Testing and Evaluating Migration/Release of Nanoparticles from Polymer Based Nanocomposites

**DOI:** 10.3390/nano10061113

**Published:** 2020-06-05

**Authors:** Roland Franz, Johannes Bott, Angela Störmer

**Affiliations:** Departement of Product Safety and Analytics, Fraunhofer Institute for Process Engineering and Packaging (IVV), 85354 Freising, Germany; roland.franz@ivv.fraunhofer.de (R.F.); angela.stoermer@ivv.fraunhofer.de (A.S.)

**Keywords:** nanomaterial, nano-additive, polymer nanocomposite, migration, release, abrasion, mechanical stress test, food contact, risk assessment

## Abstract

The use of nanoadditives in food contact materials requires risk assessment to ensure consumers’ safety. The evaluation of health risk is based on the combination of two elements: hazard and exposure. For nanomaterials (NM) used as additives in nanocomposites, the exposure is directly linked to the level of migration or release of the NM into the food. In principle, appropriate methods for experimental determination and theoretical estimation of migration are available but need diligent considerations to avoid erroneous conclusions from the measured data. We propose a comprehensive test scheme based on these methods, starting with characterization of the nanomaterial itself and when incorporated in the polymer. These data form the basis for making a decision whether migration of the NM can be excluded by migration theoretical considerations or if experimental migration testing and/or abrasion testing for mechanical release should be carried out. Guidance to and considerations for each of these steps and regarding the applicable methods are discussed. In conclusion, the results will provide a basis for risk assessment, either directly when exposure of consumers to the nanomaterials can be excluded or will be very low or, in the case of evidenced exposure, in combination with then needed toxicological data.

## 1. Introduction

The application of nanomaterials to improve or design the material properties of food contact plastics and to create new functionalities has been described in numerous publications, reviews and monographs. All these publications confirmed that nanotechnology has the potential of a key enabling technology towards beneficious innovations in the area of food packaging and they provided extensive descriptions of all aspects around the use, applications and analysis, as well as safety and risk assessment, of nanomaterials in food contact materials [1,2,3,4,5,6]. Numerous substances in nanoform or containing a nano fraction used as additives in polymer nanocomposites to improve, adapt or generate particular material properties are on the market and in wide use. Examples of such additives include carbon black, silicon dioxide, titanium nitride, nano-silver, zinc oxide, layered silica minerals (nano clays) such as bentonite, montmorillonite or kaolin but also polymeric nano-additives such as (butadiene, ethyl acrylate, methyl methacrylate, styrene) copolymers [7].

However: The use of nanomaterials (NMs) in the polymer nanocomposites for food contact is accompanied by safety concerns as to whether nanomaterials or small-sized fractions of them may migrate or be released from such types of food contact polymers into foods. This is also triggered by insufficient data availability and knowledge in nano-toxicology which would be needed to assess potential consumer exposure of nanoparticles (NP) from food contact materials (FCM) where exposure includes quantity, size and form of NP. It should be noted here that, of course, any other conventional chemical substances in non-nano form used, for instance, as a chemical modifier for the better incorporation of the NM needs to be evaluated and risk assessed. This topic is considered to fall under conventional risk assessment and is not further dealt with in this publication. As risk is the product of exposure (in our case migration from FCM) and hazard (of the NM), in the absence of sufficient information on the hazard, the key question focuses on the physical process of migration or release of nanoparticles: in short, can NPs be transferred from a polymer matrix into food?

In the published literature, many articles can be found dealing with this question by experimental migration tests on a variety of polymer nanocomposites made from different polymers and nanoadditives. In many cases, nanosilver has been used due to its ease of being sensitively and specifically measured by inductively coupled plasma mass spectrometry (ICP-MS) or single particle inductively coupled plasma mass spectrometry (sp-ICP-MS) but also other nanomaterials, such as nanoclays, metal oxides and others, have been studied. Overviews of these studies including tables with compilations of the reported migration data can be found in the recently published literature [8,9,10,11]. When reading these publications, it becomes obvious that inconsistent and even contradictive migration data and conclusions have been reported as to whether nanoparticles can be released from nanocomposites for food contact applications. In another review article published by Stoermer et al. [12], the to date published literature was critically reviewed by scrutinizing the applied experimental test procedures and analytical methods as well as data interpretations and discussions. The authors reasoned that those publications which reported the migration of nanoparticles were not conclusive as to whether the migrating species was nanoparticulate or of ionic character. Providing analytical evidence to allow distinction between solid particle release and migration of dissolved ions was found and emphasized to be crucial for proper interpretation of measured migration results. Nanosilver, which was the most frequently investigated nanomaterial, as well as some other metals are easily oxidized to ions but can re-form nanoparticles at slightly reductive conditions, e.g., under the conditions of migration testing or during sample preparation. Without validation of the findings and data interpretations, e.g., by a second analytical technique, they could be misinterpreted as nanoparticle migration. Test results from an inappropriate test design when, for instance, cut edges are in contact with food simulants was also found as a potential source to give rise for misinterpretation and wrong conclusions. In this critical review [12], any unambiguous positive proof of migrating nanoparticles was not found. Furthermore, a publication [13] dealing with mathematic modelling of nanoparticle migration from polymers indicated that nanoparticles in the size range as used in food contact polymers do not have the potential for diffusion in polymers and therefore would not be able to migrate into foods under usual packaging and food contact application conditions as long as they are fully incorporated and embedded in the polymer host matrix. The authors concluded that consumers will not be exposed to nanoparticles when the contact surface of the host polymer is intact and not altered by mechanical surface stress during application.

The objectives of this publication are to give (i) advice for considerations to be taken when it comes to the question whether or not nanoparticles migrate from a polymer nanocomposite intended for food contact, (ii) guidance on how diffusion-based migration and/or mechanical release or abrasion of nanoparticles from polymer nanocomposites can be experimentally tested and evaluated including migration-theoretical considerations, and (iii) general assistance in providing or developing an answer to the question whether a consumer can be exposed to nanomaterials from food contact polymers.

## 2. Legislative Background in EUROPE and Risk Assessment Considerations

European Framework Regulation (EC) No 1935/2004 [14] lays down the general principles for any food contact material (FCM) but does not explicitly address the use of nanomaterials (NMs) in FCMs. However, the general requirements set out in its Article 3 apply to any kind of FCM and, consistently, also include FCM manufactured with and containing NMs. European Regulation (EU) No 10/2011 [15] more specifically addresses NMs based on the following logics: due to different physicochemical properties of NMs compared to larger structured substances (recital (23)), NMs may therefore size-related toxicological profiles other than the usual bulk material. Therefore, EU No. 10/2011 clarifies that substances in nanoform may only be used if explicitly authorized and mentioned in the specifications in its Annex I (Article 9). In other words, an authorization of a substance which is based on the risk assessment of the conventional bulk material does not necessarily cover its use at nanoparticulate size and therefore (recital (27)) risk assessment of engineered nanoparticles has to be performed on a case-by-case basis. Notably, this is also applicable even when the NM is not used in the direct food contact layer (but in the middle layer) as Regulation 10/2011 explicitly excludes ‘substances in nanoform’ together with substances which are carcinogenic, mutagenic, or toxic for reproduction (CMR substances) from the functional barrier concept (Article 13, paragraph 4 (b)).

To decide whether or not ‘nano-related’ risk assessment is needed it must be clarified whether the additive of concern is a NM or not. Important support towards the answer of this question is given by EU Commission Recommendation 2011/696/EU on the definition of nanomaterial [16]. According to this definition, a ‘NM’ is a natural, incidental or manufactured material containing particles, in an unbound state or as an aggregate or as an agglomerate and where, for 50% or more of the particles in the number size distribution, one or more external dimensions is in the size range 1 nm–100 nm (in particular cases the number size fraction may be set even lower in legislation). It is very important to note that the intention of this definition of the term ‘NM’ in EU legislation is to provide solely a size-related criterion without regard to hazard or risk. For proper risk assessment of a material with a size distribution in or around the range as defined by the EU Recommendation, knowledge of the toxicological profile of the material of concern is needed. Size-related limits are not necessarily linked to biological effects which may occur in both directions from the 100 nm threshold. With other words, substances at sizes below 100 nm are not principally linked to biologically adverse effects and, on the other hand, such effects cannot be excluded for sizes above the 100 nm threshold.

Risk assessment of NMs from oral route exposure is a novel and challenging area and by far not that advanced as for conventional chemicals. In 2011, EFSA has published its ‘Scientific Opinion on Guidance on the risk assessment of the application of nanoscience and nanotechnologies in the food and feed chain’ [17] which was recently updated by an EFSA SCER cross-cutting working group [18] and published after a public consultation process [19].

It is essential to note that the risk related to the use of a NM will not only be determined by its hazard, which is determined by its chemistry, physicochemical properties and biological effects but also by the potential level of exposure for the consumer. It is generally recognized that risk is the resultant of hazard and exposure. This is of particular importance in the case of nano-additives used in plastic FCM because exposure would only occur as a consequence of NM migration or mechanical release from polymer nanocomposites into foods. Therefore, it appears to be important to establish a methodology which allows us to conclude, free of artifacts and unequivocally, on this question. Of course, as with any analytical determination, the measurement of the exposure level will be limited by the sensitivity and selectivity of available analytical methods applicable for a migration/release test. Different from conventional migrating chemical substances, where either a tiered approach is applicable for safety evaluation including defined migration limits [20] or the TTC concept [21] may be applied in cases of non-intentionally added substances (NIASs), such threshold values do not yet exist for mass-based or number-based nanoparticle size distributions of potentially migrating NM fractions.

## 3. General Approach and Overview of Test Procedures

**Figure 1:**
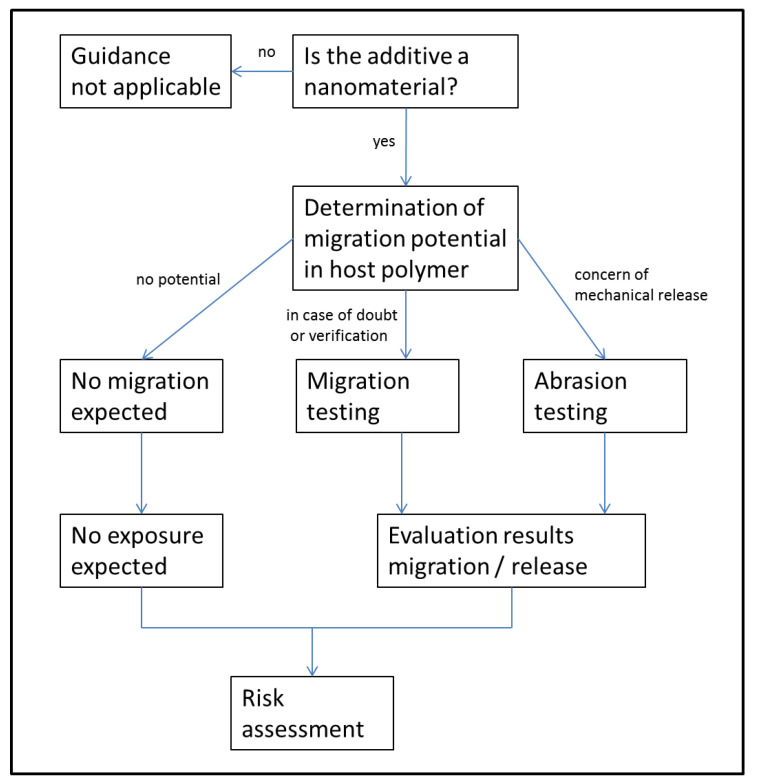
Overview of the test approach workflow.

The proposed test approach follows a decision tree (Figure 1) starting with the question whether the pure NM or additive of interest falls under the EU definition of nanomaterials [16]. Consequently, the chemical, physical and structural analysis starts with the pure additive material itself (see Section 3.1) and, in the case of positive identification as an NM, is followed by its analysis and appearance in the host polymer matrix (see Section 3.2). It should be noted here that the definition of what is a nanomaterial is arbitrary and that the proposed test approach does in principle apply to any particulate polymer additive of concern, whether or not it is technically or politically classified as a nanomaterial. In the second step, the nanocomposite is characterized and the intended applications are evaluated so that a decision can be taken either if migration of the NM or certain size fractions can be excluded by migration theoretical considerations (see Section 3.3) or if experimental migration testing with simulants or other suitable liquids should be carried out (see Section 3.4). In the case of potential mechanical release under the intended FCM use conditions of the nanocomposite, abrasion testing under appropriate material stress conditions is recommended (see Section 3.5). The subchapters advise on points to consider for suitable experimentation and theoretical evaluations. Finally, the results will assist us to perform a risk assessment either directly when the exposure of consumers to the nanomaterials can be excluded or will be negligibly small or, in the case of evidence of exposure, with consideration of the then needed toxicological data.

### 3.1. Characterisation of Additve Regarding Definition as Nanomaterial

To assist in making a decision whether or not this test approach is applicable or should be applied, as a first step it needs to be established whether the additive of concern is considered to be a NM according to the EU nano definition [16], or is likely to fall under the scope of this definition. This goes without saying that in case of doubt the approach can be applied in any case of small particles in the nano size range.

It should be noted here that identification, characterization and quantification of NM as a substance or in matrices such as polymers is a challenging undertaking and needs appropriate analytical methods not only of chemical character but also and even more of nano specific physical and material science character. In this context, very useful analytical assistance was elaborated within the EU Project FP7/2007–2013 ‘NanoDefine’. The objective of this project was to develop an integrated approach based on validated and standardized methods to support the implementation of the EC recommendation for a definition of nanomaterial. Comprehensive information, technical reports and analytical methods [22,23,24] can be found on the project’s website (http://www.nanodefine.eu/). The question as to how reliably can a material be classified as a nanomaterial, via application of particle-sizing techniques as an outcome of this project was tackled by Babick et al. [25]. Very helpful further support to this question was provided in a Science for Policy report by the Joint Research Centre (JRC) of the European Commission entitled ‘Identification of nanomaterials through measurements’ [26]. A very recently published comprehensive monography provided further in-depth scientific knowledge on the characterization of nanoparticles [27].

### 3.2. Determination of the Migration Potential of the Nanomaterial in Polymer Nanocomposites

When the additive of concern falls under the definition according to the EC Recommendation, then the potential and possibility as to whether it may migrate or be released from the host polymer of the nanocomposite into food needs to be determined and evaluated. Here, we need to consider structural differences in the application of the NM to the host or substrate polymer: is the nanomaterial homogeneously incorporated into and fully encapsulated by the host polymer, i.e., embedded with full surface coverage of the nanomaterial by the host polymer, or is the nanomaterial only applied into or onto the food contact surface where either full or partial embedment can be achieved? In the latter case, direct food contact or release from the surface may be possible or at least very likely. The migration potential, understood as the physical possibility that it may move (diffuse) within the host polymer and migrate from there into food, depends (i) on the size distribution of the free nanoparticles or aggregates in the polymer host matrix and (ii) on the degree or quality of embedment into the host polymer.

Nanoparticles with diameters larger than 5 nm are known to have extremely low diffusion coefficients in polymers [13,28] so that for usual size distributions, which do not show fractions in this low size range, migration cannot be expected under usual conditions of use as long as the host polymer fully covers the nanoadditive and encapsulates it completely. A NM incorporated into a polymer nanocomposite can be measured and characterized by transmission electron microscopy (TEM) imaging. With this technique, the distribution (homogeneity) of the nanomaterial within the polymer as well as the (predominant) particle morphology (particle/aggregate size, shape, state of aggregation/agglomeration or of exfoliation in case of clays) can be visualized and a size distribution within the polymer can be determined or at least estimated. Provided that sufficient TEM magnification is used, a conclusion on the presence of very small particles (below 5 nm) can be made which could trigger further experimental migration testing.

To clarify whether a NM is fully embedded within the polymer or (partially) protrudes from the polymer surface, electron microscopic imaging techniques like SEM can be applied to focus on the surface of the polymer nanocomposite which is intended to be in contact with the food. supplementary or even confirmatory, after the preparation of (cryo-) microtomes, TEM imaging can be used to examine the cross-section of the nanocomposite with focus on the distribution of NMs at the border region between nanocomposite and food.

The data and information obtained from these measurements are needed to support further considerations and measurement activities as described below.

### 3.3. Predictive Migration Evaluation (Based on Modelling)

The migration of organic-chemical substances such as antioxidants from food contact polymers is well understood and can be predictively calculated by use of mathematical diffusion models [29,30,31,32]. As a consequence, migration modelling was considered by EU Regulation No 10/2011 as a fast and economic tool for the evaluation of the specific migration of plastic additives: “*To screen for specific migration, the migration potential can be calculated based on the residual content of the substance in the material or article applying generally recognised diffusion models based on scientific evidence that are constructed in a way that must never underestimate real levels of migration.*” (Regulation 10/2011, Annex V 2.2.3). Therefore, based on this legislative provision, migration modelling can be applied for compliance evaluation of polymeric food contact materials, as long as the conservative nature of the migration model is ensured.

For the calculation of the migration of a polymer additive from a polymer, the migrant’s diffusion coefficient in the polymer, which can be either measured or estimated, has to be known. The key parameter to estimate the migrant’s diffusion coefficient in a polymer is its molecular volume or size which, for conventional migrants, i.e., organic chemical substances, is usually estimated by their molecular weight. The size or volume of some conventional migrants such as antioxidants is in the range of 1–2 nm diameter, which is similar to the size of very small nanoparticles. With other words, in terms of size, the distinction between very small nanoparticles and molecules is not sharp because there is an overlapping size range. From this it is hypothesized that the parameter ‘size’ of a migrant combines migration modelling of both nanoparticles and molecules and is applicable to both species. On this basis, a migration modelling approach for nanoparticles was recently developed which considers nanoparticles as quasi-molecules and, thus, enables estimating diffusion coefficients, and hence the calculation of migration values for very small nanoparticles in the diameter range up to 10 nm [13].

**Figure 2:**
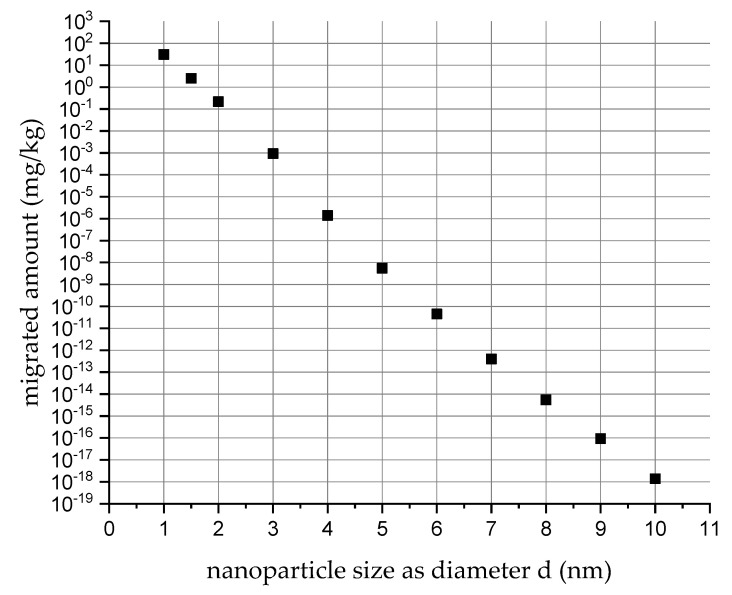
Calculated migration into food (1 kg) after contact (10 day at 40 °C) with 6 dm^2^ LDPE of 3 mm thickness as a host polymer for nanoparticles with distinct sizes from 1 to 10 nm in diameter if monomodally present each at 1000 ppm in polymer.

When taking low density polyethylene (LDPE) as a worst-case nanocomposite host polymer (from a diffusion behavior point of view), then particles with diameters from 1 to 4 nm diffusion coefficients of 3 × 10^−9^ cm^2^/s to 5.4 × 10^−20^ cm^2^/s in the polymer at 40 °C, were derived. As a hypothetical example for migration calculation, we assume that monomodal particles of these four distinct sizes are present each at a concentration of 1000 mg/kg in LDPE of 3 mm thickness. Then, after a 10 days/40 °C contact of the LDPE nanocomposite (6 dm^2^) with food (1 kg), migration values for each particle category from 30.7 ppm (mg/kg) to 1.39 × 10^−6^ ppm in food can be calculated. For a 5 nm particle size, the migration would already be as low as 5.48 × 10^−9^ ppm. For a 10 nm particle, an absurdly low migration value of 1.38 × 10^−18^ mg/kg food can be mathematically derived, which practically means “no migration”. Figure 2 shows a diagram with modelled migration values as a function of the nanoparticle size. It is emphasized that this migration scenario is for simplicity reasons based on the hypothetical presence of different nanoparticles, each monodisperse in size, to demonstrate the extremely low migration potential of nanoparticles in general. In reality, such a situation will most likely never occur due to the usually broader size distributions of the constituent particles and due to aggregation and agglomeration effects. For the modelling of such realistic situations, the amount of small nanoparticles would have to be determined by quantitative EM analysis in the polymer composite and presented as a distribution of size-specific fractions. Based on the measured concentration of each size-specific fraction, migration could then be modelled. This, however, would only be meaningful if significant mass of nanoparticles in the size range below 10 nm diameter would be present, which is very unlikely.

For polyethylene terephthalate (PET), as another host polymer with very different (much lower) basic diffusion behavior, diffusion coefficients at 40 °C were calculated for particles up to 10 nm. These values are already extremely low for 1 and 2 nm particles (1.9 × 10^−20^ cm^2^/s and 6.6 × 10^−25^ cm^2^/s). Consequently, for PET as a host polymer, diffusion coefficients for particles larger than 3 nm in diameter become meaninglessly low. Migration calculation based on these diffusion coefficients leads again to values which are extremely low, as with LDPE for sizes above 4 nm.

From this, the following important conclusion can be drawn: when it can be demonstrated that the incorporated NM has no significant fraction in the particle size range of 1–5 nm, then migration based on diffusion in the polymer will be negligibly low. As a logical consequence, it follows that when it can be demonstrated that the NM—whatever size distribution it has—is fully embedded in the host polymer matrix, then migration of the NM is not expected unless mechanical release due to material stress occurs.

### 3.4. Experimental Migration Testing

In a review of the published literature on the (potential) migration of nanoparticles from polymers, it was concluded that the reported results were largely diverging and even contradictive [12]. In most cases where positive nanoparticle migration was reported, proper experimental-analytical validation was missing and the conclusions drawn were not fully supported by the generated data. One major general drawback with the published literature is that nanosilver was the most frequently investigated nanomaterial. Due to its physicochemical properties, elemental silver can be easily oxidized into ions. This means that particulate nanosilver will not remain stable under the test conditions used but might be solubilized when the nanocomposite is in contact with inappropriate food simulants. In case of solubilization, silver ions are released, which in turn can either form precipitates (oxide, sulphide, chloride and other) in nanoform or be reduced back to elemental silver during testing and sample preparations [33,34].

In some publications, based on ICP-MS-measured silver concentrations, the conclusion was drawn that nanosilver did migrate, even when the contact medium was 3% acetic acid, in which nano silver is quickly solubilized [35,36]. Due to the considerably higher challenges in detecting and measuring the specific properties of nanomaterials compared to conventional molecular chemicals, the risk of formation of artefacts is also considerably higher, in particular when the nanomaterial is a chemical chameleon which can appear in different shapes, sizes, dissolved into ions, back-reduced into its element or chemically reacted into other species with precipitation into nanosized particles (but different from the starting NM).

When predictive migration evaluation is inconclusive or when verification of the predictive result is required, then in a next step experimental migration testing may be carried out. However, due to the inherent difficulties related to migration testing of nanoparticles from polymers, extreme care must be taken to apply a valid experimental test design and to avoid formation and measurement of artefacts. In the following, some guidance and points to consider are described when carrying out a migration test.

#### 3.4.1. Basic Considerations on Measuring Migration of Nanomaterials

Until now, there is no stand-alone technique available that is capable of detecting migration of a substance in its nanoform directly and unambiguously in a complex matrix like food or food simulants without the need for additional sample work-up or at least some fundamental thoughts that need to be considered. Depending on the chemical nature of the nanomaterial, most commonly either particle-sensitive or element-specific techniques are used in migration studies [37,38,39]. However, in contrast to migration measurements of substances on a molecular basis, proof of migration of a nanomaterial via chemical detection and identification (e.g., determination of silver as a proof of nanosilver migration) is insufficient. Besides identification and quantification, the characterization of the analyte becomes crucial to clarify whether the detected substance migrated as a nanomaterial. As mentioned above, migration testing on nanomaterials might be challenging, wherefore validation of the experiment becomes a fundamental requirement. The overall scope of the method validation is to respect the particulate nature of the NM investigated throughout the experiment to gather effects like solubilization, precipitation or interactions with other matrix components.

#### 3.4.2. Preparation of Reference Nanomaterial Dispersions

When present in a liquid matrix, substances with a particulate structure (e.g., nanosilver in food simulants) might exhibit properties differing from its solubilized form (e.g., ionic silver). To respect the physicochemical properties of nanomaterials through the experiment, fundamental steps like method development, calibration and validation should be carried out using reference dispersions. For this, the NM in the reference dispersion shall cover the same characteristics as in the nanocomposite (in sense of chemical composition and morphology). Thus, it is rather advisable to prepare individual reference dispersions by using the same nanomaterial as used in the nanocomposite investigated. Therefore, not only the amount of nanomaterial dispersed must be known, but also its particle size distribution to ensure that the reference dispersion covers the same particle sizes of the nanomaterial as present in the nanocomposite.

Shear forces applied during the production of nanocomposites (e.g., during compounding and extrusion) usually result in homogeneously and finely dispersed NMs in the nanocomposite, whereby large agglomerates are broken to smaller aggregates. When preparing NM reference dispersions the use of procedural tools (e.g., ultra-sonication bath/tip) and dispersing agents (e.g., electrostatically/sterically stabilizing surfactants, adjustment of pH-value, etc.) might be used to adjust the required properties of the NM in dispersion [40,41,42,43].

#### 3.4.3. Choice of Test Conditions for Migration Testing

Usually, test conditions for migration experiments (time/temperature/simulants) shall be chosen according to EU 10/2011. However, when nanomaterials are analyzed, the particulate nature of the analyte has to be respected throughout the experiment. Sedimentation or dissolution of the nanomaterial in unsuitable food simulants might falsify the outcome of migration experiments when the nature of the migrating substance (i.e., ionic or particulate) has to be examined. This might especially be a problem in the case of metallic nanomaterials, like nanosilver. In conventional migration testing, the choice of the more severe food simulant is based on good solubility of the analyte in the simulant. However, in the case of nanomaterials, a good dispersability without sedimentation or solubilization of the nanomaterial is required. Only that way detection of the substance in its particulate form is possible. Furthermore, good dispersibility assures the fast transport of NMs from the surface in the simulant and good capacity for the nanomaterial in the simulant, which are the characteristics for a more severe simulant. Thus, in the case of NMs, the simulant that is capable to disperse the NM best is the most severe one. However, the conventional simulants suggested in EU 10/2011 do not focus on the properties of NMs, wherefore sufficient dispersion stability with the required test conditions (e.g., 10 day at 60 °C) might not be achieved. Based on the considerations mentioned above, a dispersant solution (e.g., aqueous surfactant solution adjusted to a certain pH-value) that shows sufficient stability of the dispersed nanomaterial might be used as a more appropriate (alternative) food simulant.

As mentioned above, migration might already be excluded if a full incorporation of the nanomaterial within the polymer matrix can be demonstrated. If migration experiments are still required, only samples whose integrity can be ensured should be used. Samples that are damaged on the surface enable direct contact of the food (simulant) with NMs that are not covered by polymer anymore. Solubilization or release of “exposed surface-NMs” might falsify the results in the sense of false-positive, because diffusion-based migration did not occur [44,45]. In regard to that, special care in the preparation of migration samples must be taken. Depending on the nanocomposite that needs to be tested, the choice between single-sided contact (surface only) and total immersion of the test sample must be selected carefully. In the case of total immersion, the presence of cutting-edges exhibits an enormous risk of “exposed surface-NMs”, which might falsify the outcome of the experiment. In general, cutting-edges should be avoided and contact of the simulant with the test specimen surface only should be preferred.

#### 3.4.4. Analytical Techniques for Nanomaterials in Food Simulants

In the following, a brief summary of the most common analytical techniques used in migration studies shall be given. Element-specific techniques, like ICP-MS, find application to determine the amount of NM-specific elements in the food simulant. This technique offers high sensitivity regarding metallic nanomaterials (like silver), but does not differentiate between ionic and particulate nanomaterial species (e.g., nano-silver and ionic silver). As mentioned above, this might especially be a problem in the case of using not suitable food simulants or sample preparation (e.g., solubilisation of NMs at cutting-edges). Frequently, the use of sp-ICP-MS or the combination of ICP-MS and TEM were reported as useful techniques to clarify whether the migrated species was in its nano-form or not. Whilst TEM is suitable and recommended for the examination of the NM within the polymer host matrix (see above), examination of NMs in a liquid simulant is not possible, wherefore additional sample preparation (evaporation of simulant) is required. This is accompanied by the risk of artefact formation. Solubilized metallic nanoparticles might be reduced and re-transformed to particles/aggregates under the conditions used for TEM measurements [33]. Thus, TEM would visualize NMs though migration in its particulate form did not take place. sp-ICP-MS is able to differentiate between particulate and ionic species, but this technique suffers from more complex sample matrices [34,46]. As mentioned above, in the case of e.g., redox-sensitive silver, the formation of other silver containing particulate structures (oxides, chlorides, sulphides) might be possible, which would be detected as nanosilver by sp-ICP-MS at silver detection masses. Though ICP-MS offers excellent sensitivity for a variety of metallic elements, oxygen cannot be measured. Sulphur and chlorine show lower sensitivity than silver, which makes clear identification of the species difficult even at parallel detection.

The combination of particle-specific AF4 in combination with MALLS detection was reported to be successful in fractionation, characterization and quantification of NMs in appropriate food simulants [47]. However, this technique suffers from interferences with other matrix components of the simulant (e.g., extracted oligomers). Without separation of the NM from any other matrix components, unambiguous detection via the unspecific MALLS detector is not possible. Separation and detection via AF4/MALLS are solely based on the particle size. However, by combining the techniques mentioned before (AF4/MALLS/ICP-MS), the system is enhanced by the additional detection principle of the elemental composition of the NM [48,49,50]. That way, the separation of NMs with online characterization (particle size) and identification (chemical composition) becomes possible. By use of suitable simulants (i.e., sufficient dispersion stability and suitability for both techniques), food simulants can be analyzed directly without additional sample work-up, reducing the risk of artefact formation.

#### 3.4.5. Validation

Like in conventional analytics, validation of the experiment plays a crucial role. Detectability of the nanomaterial and avoidance of possible artefact formation must be ensured by the experimental design to avoid both false-negative and false-positive results. Validation of the experiment can be performed by using nanomaterial reference dispersions (as described above) and to consider the following points:

- Separation of other matrix components by use of fortified migration samples from the host polymer without nanomaterial (e.g., in case of less specific AF4/MALLS measurements)
- Stability of the nanomaterial during storage under migration test conditions:
  ○ test on solubilization: loss of differentiation between solubilized and particulate substances in case of element-specific measurements (e.g., metallic nanomaterial stored for 10 day at 40 or 60 °C in an acidic or aqueous simulant)
  ○ test on re-aggregation/sedimentation of the nanomaterial: loss of sample
- Influence of the analytical method and possible additional sample work up on the formation of artefacts (e.g., retransformation of ions to particles after evaporation of food simulants for TEM-measurements)

### 3.5. Consideration of Mechanical Release: Abrasion Testing

Besides diffusion-based migration, there is a risk that nanomaterials are released either due to direct mechanical impact (abrasion) and/or damaged nanocomposites surfaces caused by mechanically, thermally or chemically induced senescence of the host matrix [44,45]. In cases of concern that the food contact material undergoes mechanical stress or a potential abrasive interaction between the food and the food contact surface of a nanocomposite may occur, additional abrasion testing may be carried out. That way, facilitated migration of nanomaterials was found after stressing ceramic cookware [51] as well as polymeric cutting boards and food containers [52], whilst the unstressed food contact materials did not show migration of nanomaterials. Both studies showed that additional stress conditions that apply in practice might facilitate release of nanomaterials when the stressed materials come into contact with food again. Besides the release of nanomaterials after the stressing of the nanocomposite’s surface, the release during the mechanical stressing and thus gathering the impact of the applied stress condition itself would be of high interest. For this, we developed and tested an experimental design that allows for evaluating the release of nanomaterials during stress conditions by examining the abrasion itself on the presence of release particles. Thereby we suggested a setup that allows gathering different aspects that take influence on the integrity of the test specimen and collecting the abrasion and possible released nanoparticles without losses in a practicable way [53,54].

**Figure 3:**
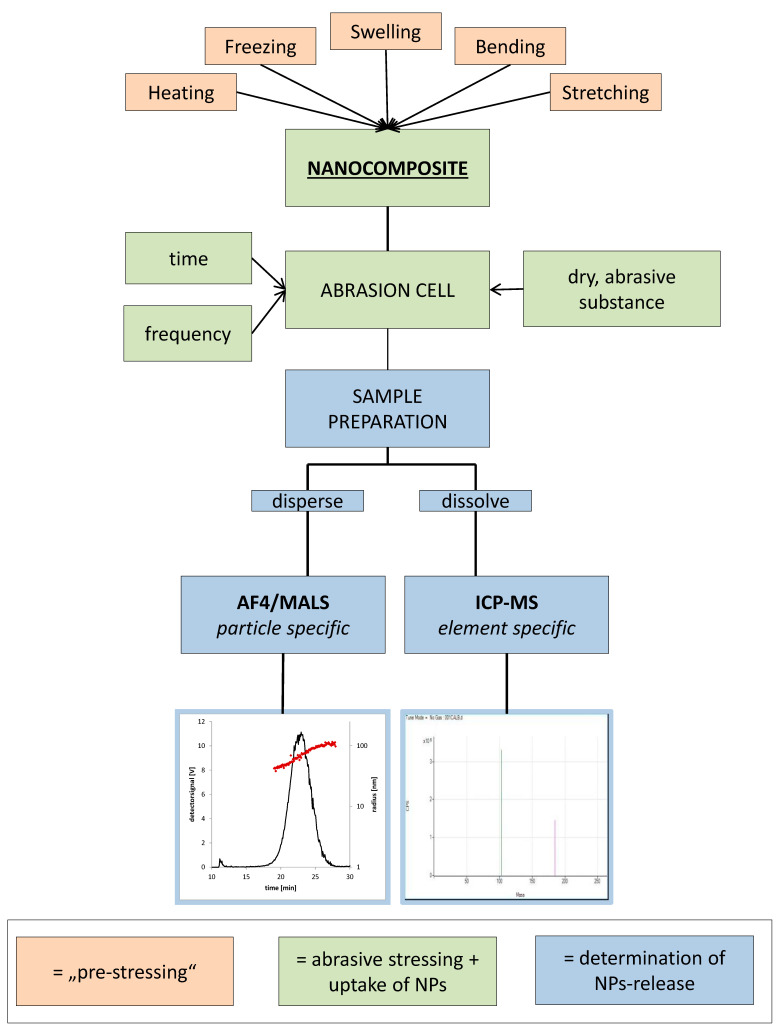
Overview workflow of abrasion testing.

The setup was already tested on a broad range of nanomaterials. In the following, a brief summary of the suggested test procedure is given (see also Figure 3). First, it is essential to simulate the foreseeable stress conditions according to the intended use and select the appropriate test conditions. The intention of this test is to stress the surface of the nanocomposites in order to evaluate whether or not mechanical impact may cause a release of NMs at or close to the surface. The setup of the stress test must therefore take influence on the surface of the nanocomposite, whilst at the same time any released NMs must be picked up and made available to detection by analytical techniques as mentioned below. The idea of the stress test is to use an abrasive substance that scrubs the nanocomposite’s surface, whereby NMs might be released (either because they protrude from the polymer or because polymer that contains nanomaterial is abraded). In a further step, the abrasive substance and the abrasion itself shall be collected completely (e.g., surface wash-off) and analyzed by suitable techniques on the presence of NMs or NM specific elements. In order to explore the general thermal, mechanical and chemical resistance of a nanocomposite FCM or to test a material for particular prefilling stress conditions, additional stressing of the nanocomposite can be performed in advance (“pre-stressing”). Pre-stressing is also recommended when stress testing and migration testing cannot be carried out at the same time. The general setup of the stress test, as schematically displayed in Figure 3, can be subdivided into three parts:

- “pre-stressing” of the nanocomposite (mechanical, thermal, solvent based)
- the actual “abrasive stress test” using dry simulants/substances with abrasive character
- analytical determination of the released NMs

The nanocomposites are clamped in a cell and loaded with a defined amount of abrasive substance (e.g., salt or quartz sand). The cells can be closed to prevent contamination from the environment and loss of sample during the stress test. (e.g., glass petri dishes). The cells can be placed on a laboratory shaker that causes the abrasive substance to homogeneously scrub over the nanocomposite surface. The intensity of this stress test can be varied by duration and the frequency of the shaking as well as by the amount of abrasive substance. Both the abrasion and the abrasive substance will then be picked up and screened on the presence of particles or particle-specific elements.

## 4. Discussion and Conclusions

The recent developments in nanotechnology have raised public safety concerns about nanomaterials in general and for consumer products with incorporated NMs particularly. This is (i) due to the fact that NMs may have different toxicological properties compared to conventional bulk material and (ii) the concern that NMs used as additives in FCM polymer nanocomposites may be released into foods under the conditions of use, thus leading to an exposure of nanoparticles to the consumer, which needs to be risk assessed. It is generally accepted that the result of any risk assessment is triggered both by the hazard of the substance under concern and the concentration of the substance in foods ingested by the consumer (exposure), i.e., the level of migration/release from the FCM. For the exposure via the oral route, it makes a crucial difference if the NM is used as a direct food additive or as an additive in a nanocomposite type of FCM from where it first needs to transfer into food before exposure can occur.

Nano additives in polymers for food packaging or kitchenware applications are usually fully incorporated into the polymer matrix and can then be considered as embedded. In these cases, migration based on Fick’ian diffusion can only happen if the embedded NM contains a fraction of nanoparticles in the 1–4 nm diameter size range. Larger particles are immobilized in a polymer matrix and do not have the potential to diffuse within the polymer to the food contact surface from where transfer into food could then occur. In cases where nanoparticles or even larger particles are not fully embedded but are protruding from and sticking out of the polymer surface, release into food under the conditions of use is not unlikely and needs to be considered for risk assessment. Such cases may occur when NMs are incorporated at very high use levels in the food contact layer or when the nanocomposite was manufactured under poor technical production conditions. Therefore, it is important to find out whether the NM is fully embedded or freely present at the food contact surface of the nanocomposite. Based on an electron microscopic imaging analysis as the first important step, it will be possible to distinguish between these situations and take corresponding follow-up measures according to the test scheme proposed above. Nanocomposites with fully embedded NM and where no very small sized nanoparticles (5 nm and smaller) can be found are very unlikely to cause exposure, unless material stress-based mechanisms such as degradation of the polymer matrix by mechanical abrasion, material fatigue processes, UV exposure, hydrolysis or swelling interactions occur under usual use conditions. It deserves a note here that in the case of nano-coatings as food contact layers on top of polymer surfaces, nanoparticulate fragments may be desorbed as a consequence of mechanical stress such as bending/stretching and/or due to weak bonding forces [38,45]. The test methods as proposed above will have the potential to also inspect these situations. It appears that one of the most important points to consider and to verify by measurements or combination of measurements is to allow clear conclusions whether the migrated or otherwise released materials are of solid, nanoparticulate character or consist of ionic species. Elemental metal determination by ICP-MS alone will not allow for drawing reliable conclusions as to whether the migrant was ionic or solid matter.

As a final summarizing conclusion, the following points should be considered for proper and conclusive risk assessment:

(1) Detailed physicochemical characterization data of the nanoadditive used, both as pristine material and when incorporated into a polymer nanocomposite (completely embedded or not), are needed.
(2) Migration/release of the nanoadditive or fractions thereof in solid form must be assessed based on the phys-chem data obtained in the first step or, if needed, on experimental data on migration/release (including abrasion) from the FCM to food.
(3) For any experimentation, it is essential to apply appropriate analytical techniques to distinguish whether migrants are in their nanoparticulate or solubilized/degraded form.
(4) When it can be shown that the migrating species are not in solid particulate form (but in ionic) or migration/release of the nanomaterial is only in trace amounts or not detectable, then nanospecific exposure appears to be negligible and risk assessment will focus on toxicological properties of the ionic species.
(5) In the unlikely case that nanoparticles do migrate or are released into foods, the established concentrations of nanoparticles in food or food simulant should be quantitatively and qualitatively characterized, i.e., the amount of particles as a function of the number-based size distribution should be determined.
(6) In the unlikely case of (5), nanospecific toxicological properties of the used nanomaterial and in particular of the small-size fraction would be required for proper risk assessment.
